# RETURNING THE FAVOR: EXPECTATIONS OF CAREGIVING RECIPROCITY AND DEPRESSIVE SYMPTOMS AMONG GRANDPARENTS

**DOI:** 10.1093/geroni/igz038.3094

**Published:** 2019-11-08

**Authors:** Cindy N Bui

**Affiliations:** University of Massachusetts Boston, Boston, Massachusetts, United States

## Abstract

This study draws upon social capital and intergenerational reciprocity concepts to better understand how grandparents’ depressive symptoms are related to their provision of grandchild care, within the context of their expectations regarding adult children reciprocating caregiving needs in the future. Analyses used the 2014 Health and Retirement Study dataset. The sample consisted of 9,612 grandparents, 2,595 of whom were providing grandchild care. Linear regression models were used to analyze how depressive symptoms were influenced by grandchild care provision and expectations of future care from adult children. Future care is measured as expectations from (1) any adult child, and (2) from the same adult child for whom the older parent provides grandchild care. Provision of grandchild care was not significantly related to grandparents’ number of depressive symptoms. Among grandparents who provided grandchild care, both expecting any adult child and expecting the same adult child were associated with reporting fewer depressive symptoms. Expecting any adult child to provide future care showed a stronger effect than expecting the same adult child to provide future care. The results suggest that expectations of general reciprocity within the family system, rather than specific dyadic reciprocity, may be more important for a caregiving grandparent’s emotional well-being. Providing grandchild care while expecting future care from adult children can indicate a sense of social capital within an intergenerational family system. Expecting support reciprocity from adult children may be a protective factor that allows caregiving grandparents to feel more secure about their future care needs, and consequently, less depressed.

